# Combining and segmenting geometric shapes into parts depending on symmetry type: Evidence from children and adults

**DOI:** 10.1177/20416695231226157

**Published:** 2024-01-23

**Authors:** Līga Zariņa, Jurģis Šķilters, Solvita Umbraško, Santa Bartušēvica

**Affiliations:** Laboratory for Perceptual and Cognitive Systems at the Faculty of Computing, 61769 University of Latvia, Riga, Latvia; Faculty of Education, Psychology and Art, 61769 University of Latvia, Riga, Latvia; Laboratory for Perceptual and Cognitive Systems at the Faculty of Computing, 61769 University of Latvia, Riga, Latvia

**Keywords:** shape, segmentation, developmental differences, features/parts, symmetry‌, spatial cognition

## Abstract

Symmetry is an important geometric feature that affects object segmentation into parts, though De Winter and Wagemans note that partly occluded objects can still be identified by the remaining visible parts. In two sets of experiments with children (*n* = 31, age 7–11, *M* = 8.8, *SD* = 1.4) and adults (*n* = 19, age 17–57, *M* = 30.4, *SD* = 12.6), we used 13 basic geometric figures distinguished by symmetry types to test how they are naturally segmented or combined and what the developmental impacts are on the segmentation and combination. In the first experiment, participants were asked to cut figures into two along a straight line; in the second experiment, participants had to create five sets of connected two-figure combinations where overlapping figures were allowed. The results confirmed the importance of the symmetry axis in both tasks. Other relevant criteria were dividing into half, maximal/minimal curvature, and use of edges or corners for reference. This study allows comparisons of the impact of symmetry type on the segmentation and combining of geometric figures and indicates developmental differences between children and adults.

What are the components of objects and shapes? How do we segment objects? How are objects combined? In this study, we are interested in the way geometric and topological objects are naturally segmented or combined and their developmental effects on segmentation and combination. Parts are functionally interdependent on the object's overall structure. Not all parts are equally important: some are functionally more important, central, and primary, whereas others are rather superficial. Functionally primary parts are those that normally trigger attention and are better encoded in memory, while others are relatively redundant for determining the object's identity. This means that objects are composed of parts that are not equally important, and objects can be bound together in particular non-arbitrary ways. Due to part–whole interdependencies, we might speculate that composing and segmenting are complementary principles in shape perception. Ultimately, our structural object knowledge enables us to encode an object's shape, relations between components that are relatively stable if compared to other components, and relations that are more superficial.

Research on object segmentation is rich, and in recent decades, several answers have been provided to such questions as what the principles of object segmentation are and what the principles of binding together objects into configurations are (for overviews, see Baker et al., 2021; De Winter & Wagemans, 2006; Singh, 2014). First, a set of basic components is used to generate the overall object shape; these basic components are computed to generate the overall shape. Prominent examples of this view are Biederman's geon approach (Biederman, 1987) and Marr's cylinders (e.g., Marr & Vaina, 1982). Geometric or other principles of perceptual organization then enable us to find parts or extract them in a shape. These principles include convexity and axial information. Burbeck and Pizer (1995) argue that object identification and representation are based on axially constrained region boundaries.

Other global shape features (e.g., proximity, symmetry, and collinearity) seem to constrain the ways objects are segmented. A somewhat special boundary-based principle of object segmentation is the minima/maxima of curvature principle (points on the contour where negative/positive curvature is extreme seem to be the most likely segmenting line). Maxima mainly applies to contour segmentation (contour as a 1D sequence of curvature values) and minima to part segmentation (parts as 2D primitive shapes) (Hoffman & Richards, 1984; for an overview and discussion, see De Winter & Wagemans, 2006, 2008; for the idea that curvature extrema induces perceived saliency, see Attneave, 1954). However, perceptual saliency is also determined by factors other than curvature extrema (e.g., points where the line segment makes a sharp turning angle) (De Winter & Wagemans, 2008).

According to Hoffman and Richards (1984), the visual system segments shapes into parts according to part boundaries instead of part shapes. Yuille and Leyton argue that shape perception is based on structural features of curvature (in particular, points where the curvature is the greatest, i.e., curvature extrema) and symmetry (Leyton, 1987, 1989; Yuille & Leyton, 1990). Additionally, based on the symmetry–curvature duality theorem (Leyton, 1987, 1989), symmetry structure and curvature extrema are linked in the way that “any section of curve, that has one and only one curvature extremum, has one and only one symmetry axis. This axis is forced to terminate at the extremum itself” (Leyton, 1987, p. 329). A more interactive model integrating both the minima rule and global shape information is proposed by Siddiqi, Tresness, and Kimia (1996), in which, according to the principle of good continuation, limb-based (negative curvature with good continuation) and neck-based parts (narrowings in the shape) are segmented.

Human subjects seem to prefer a “short-cut” segmentation based on the points that are in close proximity to one another such that the segmenting would form a part where several additional principles are taken into account (Singh et al., 1999). For instance, the connecting line must be straight and should cross a local axis of symmetry, and at least one point should be of minimal negative curvature (De Winter & Wagemans, 2006). Finally, a combination of all principles is possible as well. De Winter and Wagemans (2006) propose such a model, arguing for the preference for short lines (over long lines) in partitioning and interaction between local and global factors.

Although contours are crucial in segmenting, the overall region-encoded shape information (axis curvature, shape width, parallelity, and symmetry) also seems to be crucial; in short, according to the majority of current models, contour and region geometry seem to interact (Singh, 2014). Consistent with this thesis is a recent proposal arguing that humans segment visual shapes based on constant curvature segments that are the building blocks of shapes (Baker et al., 2021). Additionally, any natural (in a broad sense of the word, e.g., leaves, tumors, clouds, embryos) object shape seems to be linked to the object's causal history—a shape can be considered as a single state in and of a causal process (Leyton, 1989) that can further be expressed in the way it is segmented or combined. Curvature variation constrains the process that could have happened with that shape, and symmetry axes impact how a process might have changed the shape in terms of perceptual organization (Leyton, 1984, 1989).

Another prominent discussion regarding shape perception concerns the impact of symmetry if compared to elongation and reference frame structure alignment. According to the seminal work by Palmer (1983, 1985), symmetry and the reference frame constrain shape perception, and axial structure might be derived from the reference frame structure. The suggestion that elongation is primary and sufficient instead of symmetry might be significant (Sekuler, 1996; recent support of elongation is provided by Chaisilprungraung et al., 2019).

Sekuler and Swimmer (2000) provide an interactive and more inclusive approach, arguing that, in order to perceive an object's primary axis, both symmetry and elongation are used interactively (but elongation or symmetry might dominate according to its relative salience). An approach adopted prior and contrary to Sekuler and Swimmer (e.g., Quinlan & Humphreys, 1993) assumes that only symmetry (but not elongation) determines an object's primary axis (or that elongation is relatively unimportant). These differences in results might be partially due to differences in experimental settings or stimuli selection.

If these approaches are summarized, we might agree with Chaisilprungraung, German, and McCloskey (2019) that there is an elongated-part hypothesis (or set of approaches) assuming that axes are generated according to the most elongated part of an object and global shape approaches assuming that axes are generated by an object's overall shape. Notwithstanding the role of overall shape, symmetry, and elongation, there is also evidence indicating that the role of the axes of elongation and symmetry in object recognition is relatively minor if objects are rotated (Large et al., 2003).

Finally, are perceived shapes ultimately generated out of shape primitives? While physical connection supports combining and integrating parts into single objects, concavities along object contours seem to be obligatory for segmenting. Preattentive and contour discontinuities are also crucial and supportive for segmentation. However, object rotation impacts the segmenting/combining (Baylis & Driver, 1995; van Lier & Wagemans, 1998). One takeaway message from these results might be that to understand a shape's constituents and representational unity, we should be able to understand core principles and representations of the part-connections in both segmenting and combining (van Lier & Wagemans, 1998).

In general, sensitivity to symmetry seems to be a feature of human nature and might have an impact on the development of abstract knowledge in general and the structure of language in particular (Dehaene et al., 2022; Hafri et al., 2023). At the same time, people are sensitive to several other geometric properties (e.g., orientation, curvature, orthogonality, and parallel lines). It might also be the case that during childhood, core geometric principles are rapidly developed in line with Euclidean geometry and in the absence of mathematical training (Izard et al., 2011).

In this study, we are interested in whether symmetry in object segmenting and combining is developmentally sensitive and differs in different age groups. If younger children show a stronger preference for symmetry, this might indicate that symmetry is developmentally prior. Vertical symmetry is early or innate, whereas sensitivity to other forms of symmetry develops slightly later (Bornstein & Krinsky, 1985; Bornstein et al., 1981). It might also be the case that the concept of symmetry undergoes a process of developmental differentiation and that children initially have a concept of general symmetry and other (specific) symmetries are acquired later (Hu & Zhang, 2019). Although some geometric intuitions can be observed in small children, sensitivity to geometry seems to improve with age (Izard & Spelke, 2009). How exactly does the sensitivity to and perception of symmetry (and its subtypes) with respect to shape perception change in later infancy? And what are the core differences between children and adults?

Although we are particularly interested in perceptions of symmetry, differential sensitivity to several types of it, and its developmental changes, the overall framework underlying the current work is perceptual organization, focusing on how humans group items of visual input and segment shapes and objects from their backgrounds. We assume that there are part–whole principles enabling shape and object perception in general and segmenting and combining in particular. The way segmenting and combining occurs in human perception is not arbitrary but part of the general processes of shape assignment, which, in turn, happens by virtue of figure-ground segmentation, which is interrelated with grouping (for in-depth overviews, see Palmer, 2002; Wagemans, 2018; Wagemans, Elder, et al., 2012; Wagemans, Feldman, et al., 2012). A detailed analysis necessitates a different study, so we assume here that segmenting and combining support the generation of closed, simple, and symmetric structured wholes (gestalts). Moreover, our study highlights symmetry (for the gestalt-theoretic context, see Bahnsen, 1928) in ecologically valid and unconstrained situations and by using stimuli that do not directly resemble objects from everyday environments, suggesting that humans seem to prefer different principles of symmetry in the way they combine and dissect shapes (assuming the ceteris paribus condition) and taking into account developmental differences.

## Materials and Methods

We conducted two sets of experiments using 13 paper figures distinguished by symmetry types. Both experiments were conducted on-site for two groups of participants—children (*n* = 31) and adults (*n* = 19). In the first experiment, participants were asked to cut the figures into two parts along a straight line, and in the second experiment, participants had to create five sets of connected two-figure combinations where an overlap between figures was allowed.

### Participants

A total of 50 subjects participated in the study, with ages ranging from 7 to 57. The children group consisted of 31 first- to fourth-grade students aged 7–11 (*M* = 8.8, *SD* = 1.4, *Mdn* = 9), 65% of whom were girls and 35% were boys. This age corresponds to the primary school level of the Latvian education system. The adult group consisted of 19 participants, 21% male and 79% female, aged 17–57 (*M* = 30.4, *SD* = 12.6, *Mdn* = 28). Of the subjects, 90% of the children and 95% of the adult participants were right-handed. Most of the participants took part in both experiments. For those who did, the order in which they took the experiments was random.

All participants took part in the study voluntarily. Children participated with the consent of their parents. Participants were informed about the data collection policy, the opportunity to get acquainted with the results, and the possibility of withdrawing from the experiment at any time. The study was approved by the University of Latvia Research Ethics Committee in Humanities and Social Sciences (Nr. 71-46/72).

### Stimuli and Procedure

**Figure 1.**
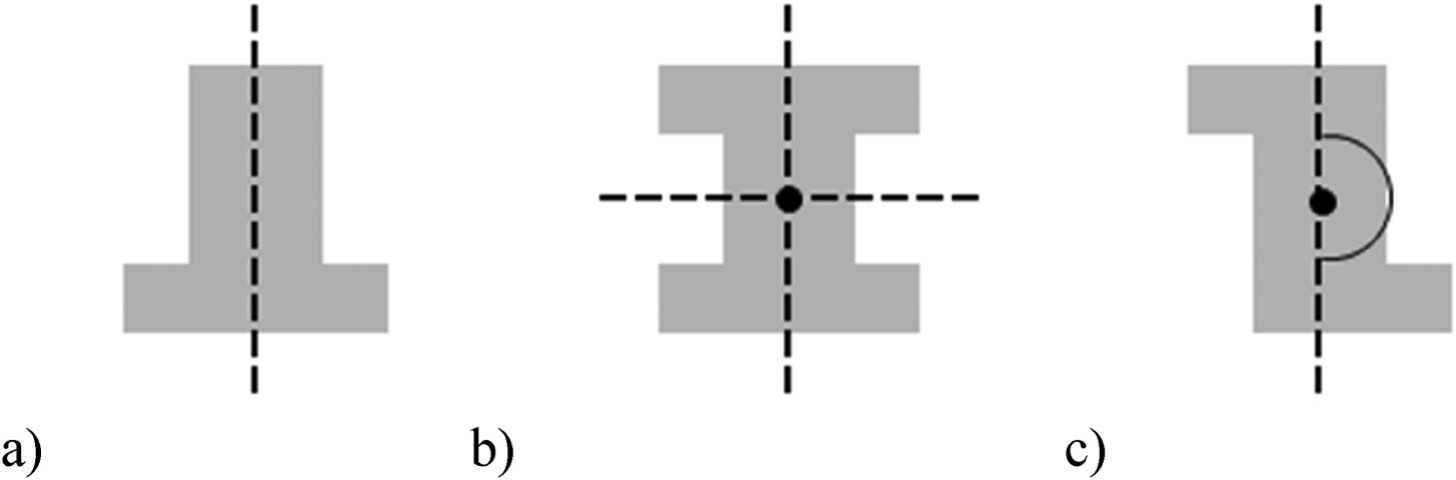
Symmetry features used in stimuli (corresponding symmetry group): (a) mirror or bilateral symmetry (D1), (b) dihedral symmetry (D2), and (c) cyclic symmetry (C2).

The stimuli for both experiments were specially designed paper figures distinguished by symmetry features. The stimuli were generated by implementing reflection and rotation symmetry. Objects with symmetry properties can be divided into separate symmetry groups (e.g., Bunch, 1989; Gruenbaum & Shephard, 1987). If an object has only reflection (mirror) symmetry along one axis, it belongs to the bilateral symmetry group D1. If, in addition to the reflection symmetry, the object also has rotational symmetry, then it belongs to one of the dihedral symmetry groups D2, D3, … Dn, depending on the number of rotation and reflection symmetry axes. If the object has only rotational symmetry, then depending on the number of possible symmetric rotation turns within 360°, the object belongs to one of the cyclic symmetry groups C2, C3, … Cn (Figure 1). C1 is the trivial group, which relates to asymmetric figures.

The figures used in the study with descriptions of their symmetry features are shown in Table 1. They include basic standard geometric shapes (e.g., triangles, quadrilaterals, ellipses, and circles) belonging to certain symmetry groups and some asymmetric shapes—one angular and three rounded (the latter were created to represent deviations from D2, C2, and D4 symmetry).

**Table 1.** Experimental stimuli with their symmetry features and proportions (height:width).

1. Circle	2. Ellipse	3. Square
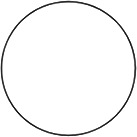	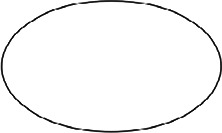	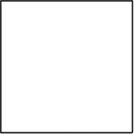
Dn dihedral rotation symmetry	D2 dihedral rotation symmetry	D4 dihedral rotation symmetry
Proportion: regular, 1:1	Proportion: 3:5	Proportion: regular, 1:1
4. Rectangle	5. Equilateral triangle	6. Isosceles triangle—Tall
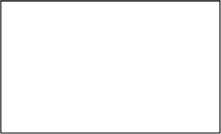	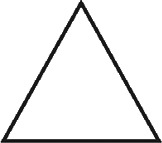	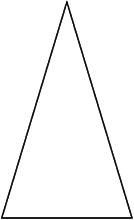
D2 dihedral rotation symmetry	D3 dihedral rotation symmetry	D1 bilateral (mirror) symmetry
Proportion: 3:5	Proportion: regular, 1:1	Proportion: 5:3
7. Isosceles triangle—Wide	8. Trapezoid	9. Parallelogram
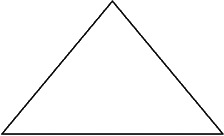	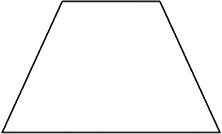	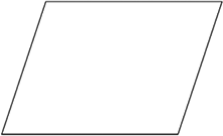
D1 bilateral (mirror) symmetry	D1 bilateral (mirror) symmetry	C2 cyclic rotation symmetry
Proportion: 3:5	Proportion: 3:5	Proportion: 3:5
10. Asymmetric—Geometric, 11. Asymmetric—1, 12. Asymmetric—2, 13. Asymmetric—3		
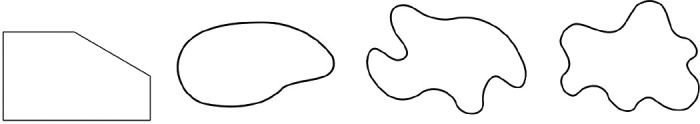		
Proportions: 3:5, 6:10, 6:10, 8:10		

We chose these figures because they are abstract, without particular context, but are, at the same time, mostly intuitively familiar to participants (except rounded asymmetrical figures). The figures’ size was equalized in terms of proportions and length of the edges (we did not consider area) and adjusted for each task (the maximal length of the edge for the first experiment was 10 cm and 5 cm for the second experiment).

To test the effects of rotation and mirror symmetry separately, we chose a parallelogram that belongs to the C2 symmetry group (rotation only by 180°) and a trapezoid corresponding to the D1 symmetry group (mirror symmetry along only one axis). The trapezoid was also included to assess the effect of parallel edges, and the two isosceles triangles corresponding to the D1 symmetry group—tall and wide—were used to explore the effect of proportion. Figures of symmetry groups including more than one symmetry axis relate to the dihedral symmetry groups D2, D3, D4, and Dn. The study included both an angular (rectangle) and a rounded (ellipse) figure with D2 symmetry to evaluate the effect of curvature. To test the impact of the number of symmetry axes, we included a regular triangle with three symmetry axes, a square with four symmetry axes, and a circle with infinite symmetry axes.

For the data analysis, we defined an orientation for each figure—horizontal, vertical, and oblique (except for the circle, for which we assumed any direction to be vertical). The figures in Table 1 are presented so that the horizontal direction is east–west (in most cases, it corresponds to the figure's longest length), the vertical is south–north, and the oblique direction is any other direction that is not horizontal or vertical. For the square, the vertical and horizontal directions match, so we assume it to be vertical; for the equilateral triangle, we take all axes perpendicular to the edges to be vertical.

### *Experiment 1*—*Cutting Figures*

The stimuli for the first experiment were figures (Table 1) made of white paper with the following dimensions—min 6 cm (height) and max 10 cm (width). The participants’ task was to cut a figure along a straight line with scissors.

The experiment began with a brief introduction and description of the task and procedure. The experimenter and the participant sat on opposite sides of the table, on which the stimuli were placed in a pile. The participant was provided with scissors and instructed, “Please cut the given figures into two parts using one straight cut” (we specifically did not say “in half”). We chose such a general and open-ended instruction because we did not want participants to be induced into choosing any specific strategy for the task. After confirming that the task was clear, participants were given 13 experimental stimuli in a randomized orientation and order.

Afterwards, the cut figures were collected, and the remaining parts were manually marked on the stimuli template forms to document the cuts. The specific features of the cuts were categorized and are summarized in Table 2. The categories are based on the shape's geometric features—(a) symmetry axis, (b) midpoints of the edges, (c) max/min curvature of curved lines, (d) angles (corner, cutting direction), (e) parallel and (f) perpendicular axes with respect to the edges, (g) half of the figure area, and (h) symmetry of the parts after the cut. Each cut was coded with “1” if it belonged to a particular category or by “0” if it did not. One cut could belong to multiple categories.

**Table 2.** Experiment 1 categories with examples from the data.

Category	Description	Examples
C1.1	Cut along the symmetry axis	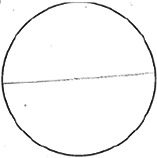
C1.2	Edge (at least one) is cut in the middle	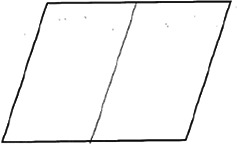
C1.3	The figure is cut in half	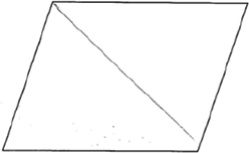
C1.4	Max/min curvature is cut (at least one)	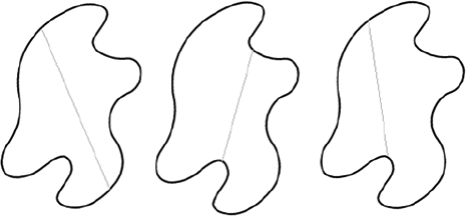
	Three types of cuts depending on the ends of the cuts: (1) max–max; (2) min–min; (3) max–min, max–other, min–other.	
C1.5	Angle is cut (at least one)	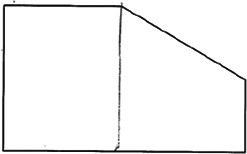
C1.6	Orientation of the cut (horizontal, vertical, oblique)	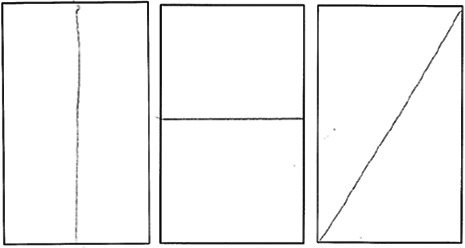
C1.7	Parallel cut with respect to the edge (at least one)	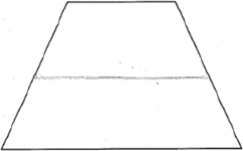
C1.8	Perpendicular cut with respect to the edge (at least one)	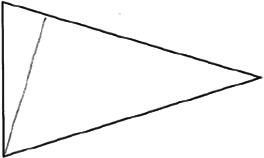
C1.9	Cut part is a corner (cutting the edges but not dividing corners)	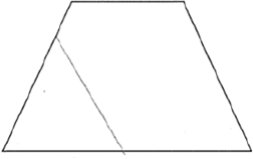
C1.10	The cut part is symmetrical (at least one)	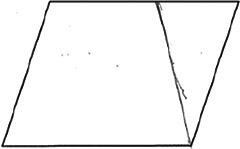
C1.11.	Arbitrary cut (does not belong to categories C1.1–5 or C1.7–10)	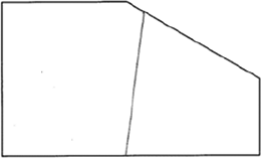

### *Experiment 2*—*Combining Figures*

The stimuli for the second experiment were made of thick gray paper with the following dimensions—min 3 cm (height) and max 5 cm (width).

The experiment began with a brief introduction and description of the task and procedure. The experimenter and the participant sat on opposite sides of the table. Before starting the experiment, we made sure that the participants understood the task and knew what was meant by the figures touching and overlapping. All the stimuli were laid out on a white table in a random order and orientation, and the participant was asked to select any two stimuli and create a combination in which the selected stimuli overlapped or touched each other. After the combination was created, the experimenter took a photo and replaced the stimuli with the others in a random order. This process was repeated five times.

Afterwards, the photos were analyzed, and the characteristic features of the combinations created were categorized according to the combinations’ topology and geometry. Figures could overlap or touch each other. If the figures were touching, the type of touch—with a point or with an edge—was determined. Regarding the geometry, it was determined whether the contact was on a symmetrical axis, the midpoint of an edge, or a corner and whether the resulting combination was symmetrical. The main categories are summarized in Table 3.

**Table 3.** Experiment 2 categories with examples from the data.

Category	Description	Examples
C2.1	Selected Figure 1	One of the figures from Table 1
C2.2	Selected Figure 2	One of the figures from Table 1
C2.3	Figures overlap or touch each other	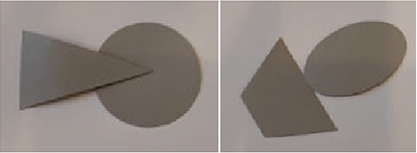
C2.4	Figures touch each other (point–point, point–edge, edge–edge)	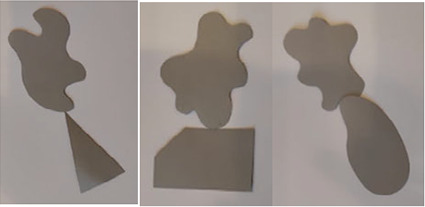
C2.5	Symmetrical combination	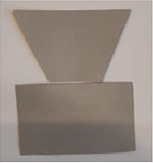
C2.6	Connection on symmetry axis (at least for one stimulus)	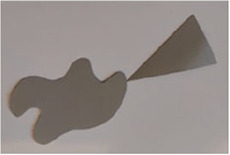
C2.7	Connection on the middle of the edge (at least for one stimulus)	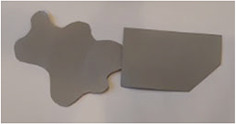
C2.8	Connection with a corner (at least for one stimulus)	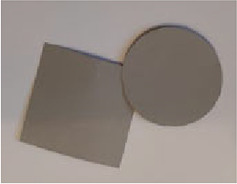
C2.9	Connection with max/min curvature (at least for one stimulus)	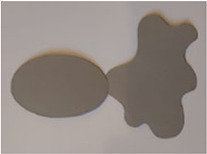
C2.10.	Matching edge length or curvature shape	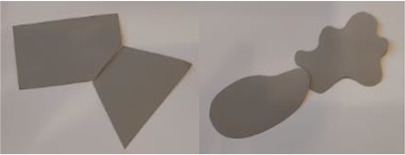

The statistical analysis (chi-square test, binary logistic regression, and multinomial logistic regression) was conducted using the software SPSS Statistics 22. The datasets generated during the study are available from the corresponding author on request.

## Results

The categories (Tables 2 and 3) were analyzed as dependent variables based on their frequency and variety corresponding to each figure (Table 1). The relative frequencies (%) are summarized in the following tables (frequencies below 5% are not included). Category C1.11, corresponding to arbitrary cuts, had a frequency below 5% for all stimuli, so this category was not included in the analysis.

We used chi-square tests to examine group (children and adults) differences within each category. To examine the effect of figure type within each category, we used binary logistic and multinomial logistic regression with the group and figure type as factors.

### *Experiment 1*—*Cutting Figures*

The symmetry axes of the figures frequently matched the cuts (particularly in the children group) (category C1.1, Table 4). A chi-square test confirmed a significant difference between children and adults in the use of the symmetry axis for the cut (χ^2 ^= 32.81, *p* < .001) (Table 4). The binary logistic regression analysis indicated a significant effect of stimuli 6 and 9 on the choice to cut the figure along the symmetry axis (Table 7).

**Table 4.** Frequencies of the cutting features related to figure geometry, %.

Stimulus	1	2	3	4	5	6	7	8	9	10	11	12	13
	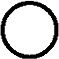	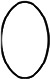	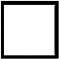	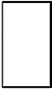	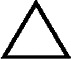	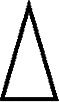	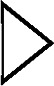	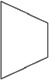	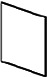	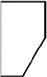	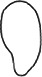	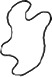	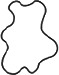
Category													
C1.1 Cut along symmetry axis ^a^													
Children	84	76	73	73	61	48	58	65	—	—	—	—	—
Adults	28	33	28	28	22	17	44	22	—	—	—	—	—
C1.2 Edge is cut in the middle ^a^													
Children	—	—	57	73	77	74	77	84	42	61	—	—	—
Adults	—	—	6	28	44	39	50	39	17	33	—	—	—
C1.3 Figure is cut in half ^a^													
Children	84	86	73	83	61	52	68	77	77	45	80	81	84
Adults	22	44	28	39	28	39	44	33	28	17	78	89	78
C1.4 Max/min curvature is cut ^b^													
Children	77	79	—	—	—	—	—	—	—	—	90	97	87
Adults	22	33	—	—	—	—	—	—	—	—	72	100	94
C1.5 Corner is divided by cutting ^b^													
Children	—	—	27	17	61	58	68	10	39	68	—	—	—
Adults	—	—	11		22	44	50	11	11	59	—	—	—

*Note.* Frequencies below 5% are not included.

a Significant relationship (α = .01) with the group according to the chi-square test. ^b^ Significant relationship (α = .05) with the group according to the chi-square test.

In some of the figures, the symmetry axis crosses the midpoints of the edges (e.g., in the case of dihedral symmetry), which was one of the typical features involved in the cut (category C1.2, Table 4). A chi-square test again indicated a significant relationship with the group (χ^2 ^= 32.08, *p* < .001): children cut edges in the middle more frequently than adults (Table 4). According to the binary logistic regression, figure type and group had a general effect, but no specific figure significantly affected the choice to cut the edge in the middle (Table 7).

Symmetric figures which had been cut along the mirror symmetry axis always were cut in half, but there were other typical situations where figures were cut in half exactly (e.g., rectangle cut along the diagonal) or approximately (asymmetric figures). A chi-square test confirmed that children cut figures in half significantly more frequently than adults (χ^2 ^= 52.07, *p* < .001) (category C1.3, Table 4). The binary logistic regression indicated an overall effect of group and figure type, with rounded asymmetric figures (stimuli 11, 12, and 13) being cut in half significantly more and the angular asymmetric figure (stimulus 10) significantly less often (Table 7).

For stimuli without straight edges, we checked how often the maximum or minimum curvature was cut. For the geometric figures (stimuli 1 and 2, Table 1), children used more cuts in this category than adults (category C1.4, Table 4), which in most cases also corresponded to cutting the stimuli in half. For rounded asymmetrical figures (stimuli 11–13, Table 1), both children and adults usually used the max/min curvature for their choice of cut (87%–90% and 72%–100% of cases, respectively). A chi-square test showed a significant (χ^2 ^= 5.20, *p* = .023) dependency of the group and used max/min curvature for cutting. The regression analysis indicated that both group and figure type, particularly asymmetric figures, had an impact on the use of max/min curvature for cutting (Table 7). To cut stimulus 11, the maximum curvature was used at both ends of the cut. To cut stimuli 12 and 13, children used the minimal curvature at both ends of the cuts (42% and 53%, respectively) more frequently than adults (16% and 22%). Adults used the maximum curve at both ends of the cuts (79% and 78%) more frequently than children (23% and 17%).

For the angular figures, we tested how often a corner was divided by cutting. Again, we found a significant dependency of the group (χ^2 ^= 9.32, *p* = .002), and regression analysis also indicated the figure type's overall effect. The summarized frequencies indicate that the corners of triangles and angular asymmetric stimulus were divided most often. In general, children cut corners more frequently than adults.

We also considered the orientation of the cut using the previously determined reference orientations (Table 5). A chi-square test showed a significant relationship in this aspect when comparing the groups of children and adults (χ^2 ^= 10.15, *p* = .006). The multinomial regression analysis showed that both group (*p* = .003) and figure type (*p* < .001) are significant. The summary of frequencies shows that children used horizontal cuts for asymmetrical figures and figures with symmetry type D4 more often than adults. In general, adults used oblique cuts more often than children.

**Table 5.** Frequencies of the cutting features related to orientation, %.

Stimulus		1	2	3	4	5	6	7	8	9	10	11	12	13
		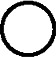	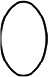	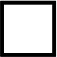	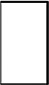	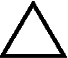	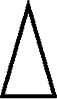	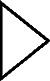	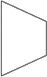	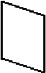	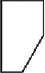	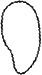	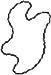	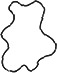
Category														
C1.6 Orientation of the cut ^a^														
Children	V	100	48	53	53	61	52	55	77	29	55	40	45	29
	H		31		20	19	23	13	6		16	33	10	26
	O		17	47	27	19	26	32	16	68	29	27	45	44
Adults	V	100	72	22	33	28	56	22	56	17	61	83	67	28
	H		6		11	39		33	11				6	6
	O		17	78	50	33	44	44	33	83	39	17	28	67
C1.7 Parallel cut with respect to an edge														
Children		—	—	53	73	19	29	26	13	16	61	—	—	—
Adults		—	—	22	50	44	33	22	11	28	57	—	—	—
C1.8 Perpendicular cut with respect to an edge ^a^														
Children		—	—	53	73	68	58	68	81	32	61	—	—	—
Adults		—	—	22	50	28	33	56	56	17	61	—	—	—

*Note.* V = vertical, H = horizontal, O = oblique.

a Significant relationship (α = .01) with the group according to the chi-square test.

Frequencies below 5% are not included.

We also tested the differences in how often the cut was made parallel or perpendicular to the edge (categories C1.7 and C1.8, Table 5). A chi-square test indicated a significant dependency of the group for perpendicular cuts (χ^2 ^= 11.48, *p* < .001), but there was no significant relationship in the case of parallel cuts (χ^2 ^= 0.90, *p* = .343). The binary logistic regression indicated a significant effect depending on the figure for both categories.

Next, we evaluated the parts that remained after the cutting (Table 6). For angular figures, we tested how often a corner was cut off the stimulus. A chi-square test indicated significant group dependence (χ^2 ^= 18.14, *p* < .001). The regression analysis also showed a significant effect of the figure (Table 7). The frequency summary indicates that adults generally cut corners more often than children. Children cut corners most frequently from triangles (32%–39%). Adults cut corners relatively more often from regular figures (square—50%, equilateral triangle—72%), as well as the wide isosceles triangle (50%).

**Table 6.** Frequencies of the cutting features related to cut parts, %.

Stimulus	1	2	3	4	5	6	7	8	9	10	11	12	13
	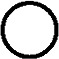	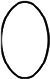	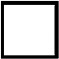	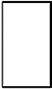	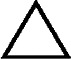	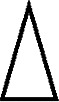	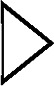	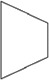	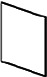	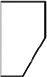	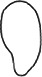	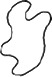	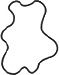
Category													
C1.9 Cut part is a corner ^a^													
Children	—	—	17	7	39	39	32		6	6	—	—	—
Adults	—	—	50	22	72	33	50	39	39	11	—	—	—
C1.10 Cut part is symmetrical													
Children	100	86	87	80	29	42	39	19	32	87	83		32
Adults	100	78	67	56	61	67	44	50	72	72	67	6	22

*Note.* Frequencies below 5% are not included.

a Significant relationship (α = .05) with the group according to the chi-square test.

**Table 7.** Significant factors according to regression analysis for figure cutting.

Category	Figure effect														Group effect	Nagelkerke *R*^2^
	Figure overall effect	Effect for specific stimulus														
		1	2	3	4	5	6	7	8	9	10	11	12	13		
		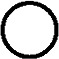	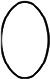	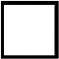	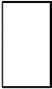	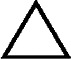	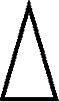	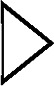	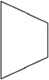	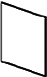	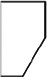	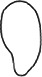	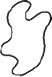	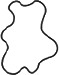		
C1.1	xxx						xx			xxx					xxx	.514
C1.2	xx														xxx	.585
C1.3	xxx										xx	x	xx	xx	xxx	.218
C1.4	xx											xx	xxx	xxx	xxx	.868
C1.5	xxx														xxx	.460
C1.6	xxx														xx	.275
C1.7	xxx															.425
C1.8	xxx														xxx	.542
C1.9	xxx														xxx	.409
C1.10	xxx															.392

*Note.* xxx = .01 significance level. xx = .05 significance level. x = .1 significance level.

Blue = less likely use of a certain category (specific figure relative to circle or children relative to adults group).

Red = more likely use of a certain category (specific figure relative to circle or children relative to adults group).

Categories: C1.1 Cut along symmetry axis, C1.2 Edge is cut in the middle, C1.3 Figure is cut in half, C1.4 Max/min curvature is cut, C1.5 Angle is cut, C1.6 Orientation of the cut, C1.7 Parallel cut, C1.8 Perpendicular cut, C1.9 Cut part is a corner (cutting the edges but not dividing corners), C1.10 Cut part is symmetrical.

Finally, we evaluated whether at least one of the parts after cutting was symmetrical or approximately symmetrical (for asymmetric figures). Table 6 shows that it was typical to obtain a symmetrical part for most stimuli after cutting. A chi-square test did not indicate a significant dependency of the group (χ^2^ = .482, *p* < .487). However, some trends can be noticed. After cutting triangles, adults obtained symmetrical parts more frequently than children. This was also the case with the trapezoid (D1 symmetry type) and the parallelogram (symmetry type C2). Children, on the other hand, cut somewhat symmetrical parts from asymmetrical figures a little more often than adults. They also obtained symmetrical parts more often after cutting figures with symmetry types D2 and D4 (stimuli 2–4). The regression analysis confirms no effect depending on the group but shows a significant effect depending on the figure.

### *Experiment 2*—*Combining Figures*

The frequencies of selected figures for all combinations are summarized in Table 8. A chi-square test indicates no relationship between the group and the figure selected (χ^2 ^= 4.88, *p* = .962). This means the choice of figures can generally be considered similar for children and adults. Based on the frequencies, figures with well-detectable mirror symmetry were chosen most often from among the symmetric figures—trapezoid, high isosceles triangle, rectangle, ellipse, and circle with ideal symmetry (15%–22%). At the same time, asymmetric figures (stimuli 12 and 13) were selected with similar frequency (17%–19%).

**Table 8.** Selection frequency of stimuli, %.

Stimulus	1	2	3	4	5	6	7	8	9	10	11	12	13
	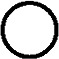	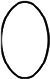	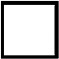	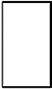	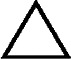	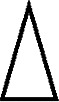	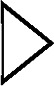	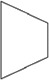	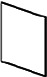	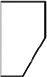	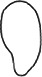	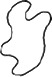	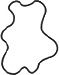
Children	16	17	11	18	9	15	15	22	17	10	16	17	17
Adults	19	17	14	21	10	21	11	16	14	10	10	19	17

In the experiment, participants could freely choose how to combine the figures by making them overlap or touch. Adult participants combined figures by overlapping them more often than children (44% and 30%, respectively; see Table 9). This difference is significant according to a chi-square test (χ^2 ^= 4.07, *p* = .044). A chi-square test indicated that the figure selected had a significant effect on the type of connection (χ^2 ^= 26.26, *p* = .010). The binary logistic regression analysis confirmed that both the figure selected and the group did have a significant effect on combination type (Table 11).

**Table 9.** Topology of the combined figures, %.

Category		Children	Adults
C2.3 Figures overlap ^a^		30	44
C2.4 Figures touch each other	Point–point	15	28
	Point–edge	15	18
	Edge–edge	70	55

a Significant relationship (α = .05) with the group according to the chi-square test.

The type of touching (point-to-point, edge-to-point, or edge-to-edge) showed no significant association with the group according to a chi-square test (χ^2 ^= 3.74, *p* = .154). Table 9 shows, however, a tendency that children combined stimuli by connecting edges more frequently than adults (70% and 55%, respectively), and adults combined figures with a point-to-point connection more often than children (28% and 15%, respectively). A chi-square test indicated that the figure selected had a significant effect on the type of touching (χ^2 ^= 70.39, *p* < .001). This was confirmed by a multinomial logistic regression analysis, which indicated that the choice of figure had a significant effect on the type of touching (χ^2 ^= 78.99, *p* < .001) and which also indicated a significant effect of group (χ^2 ^= 10.03, *p* = .007) (Table 11).

Table 10 summarizes the categories describing the symmetry features of the combinations created in each experimental group. A chi-square test indicated that adults tended to form symmetrical combinations more often than children (χ^2 ^= 2.73, *p* = .098).

**Table 10.** Characteristics of the figure combinations created, %.

Category	Children	Adults
C2.5 Symmetric combination ^a^	29	40
C2.6 Connection on the symmetry axis	74	76
C2.7 Connection on the middle of the edge	59	67
C2.8 Connection with corner	10	16
C2.9 Connection with max/min curvature	52	46
C2.10 Matching edge length or curvature shape	38	29

a Significant relationship (α = .1) with the group according to the chi-square test.

Although the frequencies of other categories implying the use of symmetry and shape regularities (connection on the symmetry axis, connection on the midpoint of the edge, and connection with the corner) are higher for adults, a chi-square test indicated no significant associations between each category and group (χ^2 ^= 0.10, *p* = .753; χ^2 ^= 1.17, *p* = .280; and χ^2 ^= 1.55, *p* = .213, respectively). Also, there were no significant associations between group and max/min curvature and matching edge length or curvature shape (χ^2^ = .70, *p* = .404 and χ^2^ = .95, *p* = .329, respectively).

Regarding the stimulus’ impact, a chi-square test indicated significant effects for connection on the symmetry axis (χ^2 ^= 157.39, *p* < .001) and the middle point (χ^2 ^= 90.07, *p* < .001), as well as max/min curvature (χ^2 ^= 169.73, *p* < .001) and matching edge length or curvature shape (χ^2 ^= 47.93, *p* < .001) but not for the connection with a corner (χ^2 ^= 17.29, *p* = .139). A binary regression analysis indicated that both the group and figure affected whether the created combination was symmetrical (*p* = .019 and *p* < .001, respectively). As for the connection on the symmetry axis, midpoint of the edge, max/min curvature, and matching edge length or curvature shape, the binary logistic regression confirmed that the group had no significant effect but that the figure did. According to the regression analysis, the connection with a corner was associated neither with the group nor the figure (Table 11).

**Table 11.** Significant factors according to regression analysis for figure combining.

Category	Group effect	Figure effect	Nagelkerke *R*^2^
C2.3 Figures overlap or touch each other	xxx	xx	.111
C2.4 Figures touch each other	xxx	xxx	.335
C2.5 Symmetric combination	xx	xxx	.372
C2.6 Connection on the symmetry axis		xxx	.479
C2.7 Connection on the middle of the edge		xxx	.284
C2.8 Connection with corner			.101
C2.9 Connection with max/min curvature		xxx	.503
C2.10 Matching edge length or curvature shape		xxx	.163

*Note.* xxx = significance level .01. xx = significance level .05.

Finally, using binary logistic regression, we examined the effect of other combination characteristics (connection type, type of touch, connection on the symmetry axis, connection on the middle of an edge, and connection with a corner, max/min curvature and matching edge or curvature shape) on the creation of symmetric combinations. The analysis showed that matching the edge or curvature shape had a significant effect on the creation of a symmetrical combination (*p* = .003, Nagelkerke *R*^2^ = .594). This category was less likely associated with symmetrical combinations.

In both tasks, symmetry affected the decision of how objects were segmented or combined, which is reflected in its frequent use. Depending on the figure, adults used the symmetry axis for cutting in 17%–44% of cases and children in 43%–81% of cases, significantly more than adults. When combining figures, the connections on the symmetry axis were 70% of the time among children and 76% of the time among adults, which is slightly more, but not significantly different. After cutting, at least one of the remaining parts was usually symmetrical for almost all figures. The groups did not differ significantly (19%–100% of cases for children, 22%–100% of cases for adults), although there were some differences in trends depending on the figure. On the other hand, when performing the combining task, the combinations made by adults were symmetrical significantly more often than those made by children (40% of cases for adults, but only 19% of cases for children).

Another important feature was the use of the midline. When cutting figures, children used the midpoint of the edge in 30%–51% of cases, depending on the figure, and the figures were cut in half in 41%–86% of cases. Adults showed greater variation across figures—they used the midpoint of the edge 17%–50% of the time and cut the figure in half 17%–89% of the time, depending on the figure, significantly less than children. In figure combinations, the connection was located at the midpoint of the edge more often among adults (67%) than children (57%), but the difference was not statistically significant.

Corners and maximum/minimum curvature were also used as a reference for cutting. Here, again, the groups of children and adults differed. Children cut a circle and an ellipse at maximum curvature significantly more often (76% and 78%, respectively) than adults (22% and 33%, respectively). Children also divided one of the corners in 11%–62% of cases, depending on the figure (for adults, corners were divided in 11%–50% of cases). Rounded asymmetric figures were cut similarly—all participants predominantly used maximum or minimum curvature (86%–95% of cases among children and 72%–100% of cases among adults), and it was used equally often when creating combinations (53% of cases).

Other differences in cutting concerned the cut's orientation, where different trends depended on figures and their symmetry. In the combination task, we found no significant differences between the groups regarding the touching or overlapping of the figures, but the figure type had an effect. Regarding the type of touching, children connected an edge with the edge more frequently (69% of cases compared to 55% of cases among adults), while adults connected one point with another point relatively more frequently (28% of cases compared to 14% of cases among children). However, the differences between groups were not statistically significant.

## Discussion and Conclusions

Our study indicates some fascinating developmental differences between children and adults in segmenting and combining tasks. Regarding symmetry, children used the symmetry axis more frequently for dividing, whereas adults created symmetric combinations more frequently. The middle point was used significantly more often by children in the cutting task, whereas it was used more frequently by adults in the combining task. In general, children and adults differed more when cutting figures than when connecting them.

When combining figures, symmetry supports single-object perception; therefore, when different figures are put together, the most symmetrical interpretation seems to be preferred (Bertamini et al., 2018). In our data, we see that even though combinations were symmetrical in less than half of cases (29% among children, 40% among adults), symmetry was involved in creating combinations in most cases (the connection was on the axis of symmetry in 70% of child combinations and 76% of adult combinations).

Our results also seem to indicate that segmenting works as simplicity generation—complex shapes are segmented into parts with less negative curvature (Singh, 2014). The importance of maximal and minimal curvature for segmentation (Hoffman & Richards, 1984; Siddiqi et al., 1996) is reflected in the cuts of rounded asymmetrical figures. Cuts of limb-like parts (Siddiqi et al., 1996) were rare, but we were able to observe the tendency to cut the shortest distance (Singh et al., 1999) in cases of rounded asymmetrical figures and figures with symmetry type D2 (rectangle and ellipse). At the same time, other geometric features (parallel edges, symmetry) were crucial for segmenting, and some figures (e.g., triangles, parallelogram) seemed to have a stronger impact. This was also found by De Winter and Wagemans (2006).

Based on our results, we also can agree with Baker et al. (2021) that shape operations, both in segmenting and combining, are relational (shape information is derived from a larger relational whole or an object's inherent parts), more economical (shapes are more economical than a random set of elements) and fulfills perceptual input (something incomplete is made better; gaps are completed). This is reflected in the frequent use of the edge-to-edge connection type and overlapping, as well as in symmetry, which is typical of cut parts. Most likely, shapes and their segments corresponding to reflectional symmetry are perceived more easily and effortlessly, which, in turn, explains the preference for the vertical axis (Bertamini et al., 2018).

The current study has several limitations. First, only abstract (geometric and topological) shapes were used, minimizing direct effects from previous knowledge and semanticity (which might also be beneficial to avoid the impact of previous experience). In future studies, other, more complex stimuli could be included to test the effect of symmetry versus maximal curvature, symmetry versus minimal distance, symmetry versus matching the length of an edge, and symmetry versus area. The geometric versus functional effects (based on previous experience and interaction) could also be tested.

The set of stimuli was created based on bilateral and rotation symmetry features. As such, stimuli containing other symmetries—translation and glide reflection—could also be included in future studies. Besides primarily perceptual features, we should also consider knowledge-driven top-down impacts. Although our stimuli are abstract and do not contain experiential features linked to everyday contexts, we might imagine that possible points for grasping, center of mass, or balancing points may be of some significance (Firestone & Keil, 2016).

Additionally, a straight line is sometimes not the most intuitive one for segmenting objects (Rosin, 2000). Finally, the sample size is relatively small for an experimental study. However, if it is considered an exploratory study, the results might be sufficient to distinguish between the main geometric categories characterizing the segmentation and combination of basic geometric figures and point to several pronounced differences between adults and children.

Although this research topic requires further study, we agree that segmenting and combining are driven by the principles of perceptual organization. Segmenting is supposed to generate two perceptually good wholes, and combining should generate a single, intuitively as good as possible whole. In both cases, part–whole principles seem to apply when inducing the best two wholes (when segmenting) and the best single shape (when combining). Finally, our results seem to support the gestalt-theoretic work on perceptual organization. For example, segmented shapes and combined ones fit into the idea of best, closed forms, good continuation, simplicity, and symmetry (Wagemans, 2018). However, the impact of symmetry, as our study shows, changes according to age and is weaker among adults.
